# Drug-resistant epilepsy in an autosomal recessive copper metabolism disorder from SLC33A1 mutation

**DOI:** 10.1016/j.ebr.2026.100894

**Published:** 2026-09-11

**Authors:** Raunak Singh, Cheng Yen Kuo, Shelja Sharma, Brian Nils Lundstrom, Donnie Keith Starnes

**Affiliations:** Department of Neurology, Mayo Clinic, Rochester, MN, United States of America

**Keywords:** SLC33A1 mutation, Epilepsy, Drug-resistant epilepsy, Neuromodulation, Vagus nerve stimulation, Pediatric neurology, Huppke-Brendel Syndrome, Copper metabolism disorder, Developmental Epileptic Encephalopathy

## Abstract

SLC33A1 mutation (Huppke-Brendel Syndrome) is a rare, autosomal recessive disorder associated with copper metabolism and characterized by global developmental delay, sensorineural hearing loss, congenital cataract and generalized hypotonia. We report two siblings under 6 years with novel SLC33A1 gene mutation, born from heterozygous parents. The first patient developed early onset drug-resistant epilepsy (DRE) secondary to the novel genetic mutation; the second patient did not develop seizures but shared several other phenotypic features of the syndrome, highlighting intrafamilial variation in seizure penetrance. On electroencephalography of the first patient with DRE, independent spike and wave discharges were noted in the temporoparietal region with generalized background slowing. Diffuse electrodecrement with overriding paroxysmal fast activity was correlated with seizure semiology. Neuroimaging revealed generalized cerebral and cerebellar atrophy, ex-vacuo ventriculomegaly, and bilateral chronic subdural collections. We describe a shared genetic mutation in two siblings and highlight their overlapping and distinct clinical features, management challenges, and complications with invasive neuromodulation. These cases expand the differential diagnosis of copper-related metabolic disorders.

**Practical implications:**

Variable seizure penetrance in SLC33A1 related disorders could manifest as epileptic phenotypes.

## Highlights

• HBS can present DRE in a patient with metabolic disorder secondary to genetic and severe neuroanatomical defects.
• This case expands the differential diagnosis of copper related metabolic disorders and resembles Menkes-like presentation.
• Novel mutation site of the genetic defect.
• Significant neuroanatomical defect and EEG features reflect epileptogenesis.
• Management of epilepsy with invasive neuromodulation devices like VNS is challenging due to complex systemic comorbidities.

## 1 Introduction

Huppke-Brendel Syndrome (HBS) is an extremely rare autosomal-recessive neurodevelopmental metabolic disorder. The index patient [1] was described in 2005, characterized by low serum copper and ceruloplasmin, congenital cataract, severe muscular hypotonia, global developmental delay, sensorineural hearing loss [1], [2], [3], [4], [5]. Currently, thirteen patients (twelve pediatric, one adult) have been reported [3], [5].

Disorders of copper metabolism include Wilson disease [6], [7], Menkes disease [6] and Aceruloplasminemia [8]. Loss-of-function mutation in the SLC33A1 gene on the long arm of chromosome 3 [2], leading to reduced or absent expression of acetyl-CoA transporter-1 (AT-1) [4], [5], [9], has been implicated in the pathogenesis of HBS. Copper supplementation shows limited effectiveness based on prior reports [1]. Offspring of heterozygous recessive parents have a 25% chance of leading to homozygous SLC33A1 variants [3].

Seizures have been reported in some patients [5], [10]. We discuss EEG and imaging features of the first patient who developed drug-resistant seizures in the first two years of life. The second patient did not experience epileptic activity but presented with similar neurodevelopmental problems and anomalies on imaging. This series expands the differential diagnosis of copper related metabolic disorders.

## 2 Case series

### 2.1 Shared genetic and clinical profile of siblings with SLC33A1 mutation

Both patients were born at term from heterozygous carriers of SLC33A1 mutation. Genomic sequencing showed homozygous SLC33A1 deletion at cDNA position 1304 in both siblings, causing a frameshift from position 435 resulting in a stop codon 14 positions downstream (c.1304del, p.Phe435Serfs*15), and subsequently a truncated acetyl-CoA transporter 1 (AT-1) protein. This novel variant falls within the C-domain (TM7–12) of AT-1, which harbors the acetyl-CoA binding site [11]. There was no family history of HBS.

Both patients were under 6 years of age at the time of assessment and shared several clinical features including global developmental delay, bilateral aphakia, bilateral sensorineural hearing loss treated with cochlear implants, bilateral congenital dysplasia of hip, recurrent respiratory and urinary tract infections, feeding difficulties, severe nocturnal hypoxemia, hypothyroidism requiring levothyroxine and iron deficiency. Both patients were generally hypotonic with variable reflexes. Most of these features were identified within the first year of life in both patients. No other dysmorphic features were noted. Both had low serum copper (<10 μg/dL) and ceruloplasmin (<7 mg/dL). Copper supplementation was not trialed in either patient due to limited benefits. Both patients followed up for about 3 years.

Case 1 Drug-resistant epilepsy in SLC33A1 mutation.

The first patient was assessed at the age of 5 years. He experienced spontaneous seizure onset before the age of 18 months. Initial seizure semiology was described as behavioral arrest, upward eye deviation, staring, unresponsiveness and bilateral leg shaking, managed with levetiracetam. The frequency gradually increased to multiple episodes per day, each lasting up to five minutes and occurring in clusters. Eventually myoclonic-tonic seizures consisting of left arm flexion and right arm extension emerged. There was significant cognitive decline after seizure onset. He was treated with prednisone for suspected epileptic spasms, based on clinical semiology and EEG findings consistent with hypsarrhythmia, with partial seizure reduction; however, this was complicated by secondary central adrenal insufficiency subsequently managed with daily hydrocortisone.

Additionally, he had cerebral palsy, severe GERD (post-Nissen's fundoplication, on orogastric tube support), low bone mineral density (*Z*-score = −2.0). He had undergone multiple surgical procedures including excision of the bilateral submandibular glands and parotid duct ligation bilaterally.

The seizures were refractory to multiple medication trials, including topiramate and clobazam. Prednisone and valproic acid were thought to be helpful, but adverse effects included infections and hyperammonemia, respectively. Ketogenic diet was started and reduced seizure frequency by approximately 40% but was stopped due to poor tolerance. Eventually he was implanted with a vagal nerve stimulator (VNS) at the age of 5 years; however, the device had to be explanted within a month post-implant secondary to wound infection. Seizure frequency at the last follow-up was 10–15 seizures per day despite treatment with vigabatrin, levetiracetam and clobazam.

Prolonged video-EEG recording (Fig. 1) demonstrated clusters of brief asymmetric myoclonic-tonic seizures with abrupt bilateral upper and lower extremity extension and stiffening and abundant independent bilateral temporo-parietal spike and wave discharges. There were numerous clusters every few hours, with several seizures in each cluster. These events were associated with diffuse electrodecrement with overriding paroxysmal fast activity (PFA). The background was normal. MRI brain (Fig. 2) revealed global cerebral and brainstem volume loss, moderate ex-vacuo ventriculomegaly, and an arachnoid cyst overlying left peri-sylvian fissure. Additional findings included falcine agenesis, cerebellar atrophy, vermis retroversion and fourth ventricle enlargement, bilateral chronic subdural hematomas likely reflecting underlying cerebral volume loss, thalamic and pontine signal abnormalities, and diffuse white matter gliosis. Imaging was limited by cochlear implant artifacts. CSF results were unremarkable except for elevated pyridoxal 5-phosphate (74 nmol/L) of uncertain clinical significance.

Case 2 Non-epileptic phenotype in SLC33A1 mutation.

**Fig. 1:**
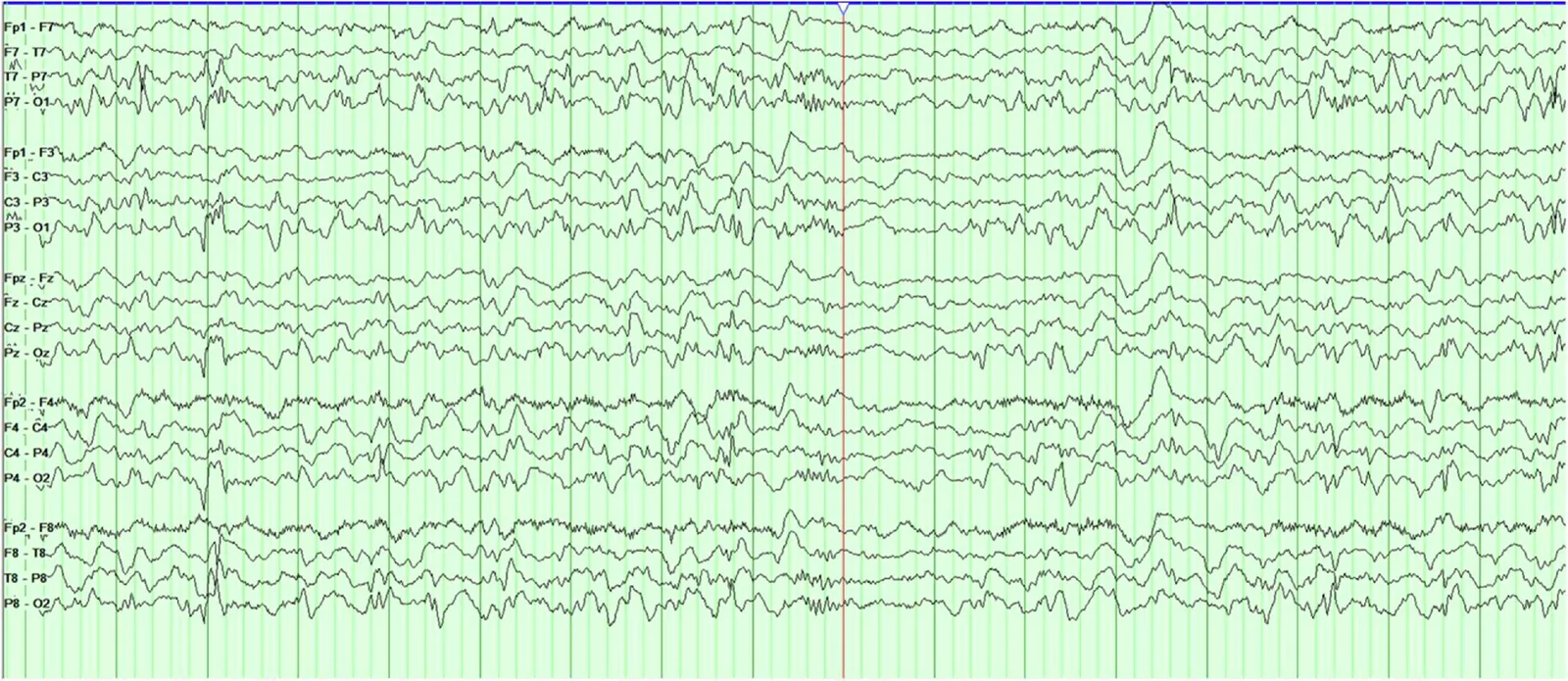
EEG findings of the first patient with DRE showed independent spike and wave discharges (1.5-2 Hz) noted in the temporoparietal region (right more than left) with generalized background slowing. Diffuse electrodecrement with overriding paroxysmal fast activity (PFA) was correlated with typical seizure semiology lasting 2–3 s. Longitudinal bipolar montage (prolonged video EEG study).

**Fig. 2:**
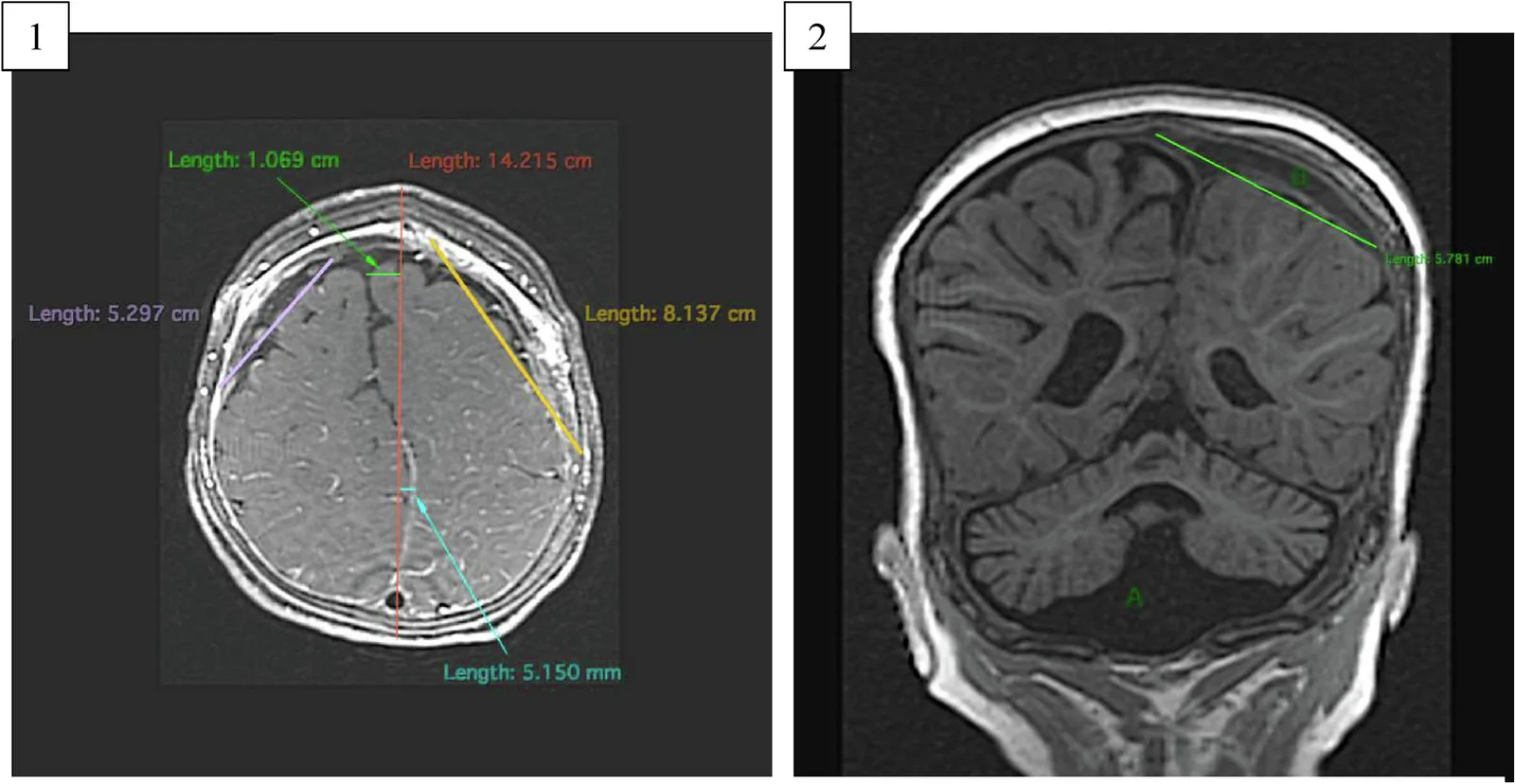
Imaging of the first patient with DRE: 1) Contrast Enhanced MRI Axial section showed anterior (1 cm), posterior (5 mm) asymmetry, anterior falcine agenesis, and crossing of left frontal lobe across the midline. Bilateral chronic subdural hematoma (8 cm left, 5 cm right). 2) MPRAGE Coronal section showed substantial global hypotrophy and ex-vacuo ventriculomegaly. A) Cerebellar atrophy and 4th ventricle enlargement. B) Chronic subdural hematoma (5 cm right).

The second patient was assessed at 13 months of age and presented with multiple comorbidities including respiratory and urinary tract infections like his sibling but did not develop seizures at the time of presentation. The EEG was normal. Brain MRI revealed apical orbital stenosis with kinking of intra-orbital optic nerves bilaterally, asymmetric calvaria which may have led to multiple cortical venous collaterals with corkscrew appearance suggestive of venous congestion, interval increase in subarachnoid space over frontotemporal convexities and a resolving left cephalhematoma. Comprehensive evaluation for respiratory distress revealed an asymptomatic patent foramen ovale (left-to-right shunt), tracheomalacia and bronchitis. He underwent multiple surgical procedures and intensive care management like the older sibling.

## 3 Discussion

AT-1 is a multi-pass membrane transporter that imports acetyl-CoA into the endoplasmic reticulum lumen, providing substrate for acetylation of ER proteins [12]. Pathogenic SLC33A1 mutations are linked to two distinct disorders: autosomal recessive HBS and autosomal dominant hereditary spastic paraplegia type 42 (SPG42) [13]. We present two cases of HBS in siblings, one of whom had severe DRE.

Though seizures have been reported in HBS, detailed semiology and neurophysiological characterization remain sparse in the literature. The sole prior report of DRE in HBS described seizure onset at 14 months with burst suppression on scalp EEG, managed with three antiseizure medications [10]. Our first patient extends this picture considerably, as we describe a comprehensive seizure history alongside characteristic EEG findings, significant neuroanatomical anomalies, and a challenging treatment course across multiple drug trials and dietary therapy. Despite these efforts, seizures remained refractory, and VNS was pursued as a palliative option [13], [14]. Unfortunately, the device required explantation due to wound infection, a complication likely compounded by ongoing immunocompromise and systemic frailty. In contrast, the younger sibling harbored the same genetic defect and shared significant comorbidities yet had not developed seizures at the time of evaluation, underscoring the intrafamilial variability in seizure penetrance that may characterize this syndrome.

These two siblings shared core functional impairments of HBS by two years of age, yet exhibited discordant imaging and EEG features, findings that contribute to the emerging literature on variable phenotypic penetrance in this syndrome. Both patients required extensive multidisciplinary care, reflecting the broad systemic burden of the disease.

HBS carries a grave prognosis, with lethal outcomes reported by six years of age in most cases, with the notable exception of a patient identified in adulthood [3]. The clinical trajectory of our patient showing early-onset DRE, characteristic EEG features and severe neuroanatomical abnormalities raise the possibility that HBS may manifest along the spectrum of developmental and epileptic encephalopathy (DEE) in some individuals [15].

## 4 Conclusion

HBS is a rare but clinically important genetic disorder of copper metabolism with a consistently severe multisystem phenotype and poor prognosis. We describe two siblings sharing a pathogenic SLC33A1 mutation who exhibited discordant neurological phenotypes, one developing early-onset drug-resistant epilepsy with features consistent with a developmental and epileptic encephalopathy, while the other had no seizures at the time of evaluation. This intrafamilial variability highlights the variable penetrance of epilepsy in HBS. We further describe the neurophysiological and neuroanatomical correlates of epileptogenesis in this context and underscore the challenges of invasive neuromodulation when systemic comorbidities are severe. These cases expand the phenotypic spectrum of HBS.

## Key points

• HBS can present with early-onset drug-resistant epilepsy with features of a developmental and epileptic encephalopathy, a phenotype not previously described in this disorder.
• Intrafamilial variability in seizure penetrance can occur in HBS even with an identical pathogenic SLC33A1 mutation.
• Severe neuroanatomical abnormalities and characteristic EEG findings likely contribute to epileptogenesis in susceptible individuals.
• Invasive neuromodulation for DRE in HBS carries high risk due to complex systemic comorbidities, including immunocompromise and systemic fragility.
• HBS should be considered in the differential diagnosis of copper-related metabolic disorders presenting with DEE, expanding the known phenotypic spectrum of SLC33A1-related disease.

## CRediT authorship contribution statement

**Raunak Singh:** Writing – review & editing, Writing – original draft, Methodology, Investigation, Formal analysis, Conceptualization. **Cheng Yen Kuo:** Writing – review & editing, Writing – original draft, Conceptualization. **Shelja Sharma:** Writing – review & editing, Methodology, Investigation, Conceptualization. **Brian Nils Lundstrom:** Writing – review & editing, Supervision, Investigation, Conceptualization. **Donnie Keith Starnes:** Writing – review & editing, Supervision, Investigation, Conceptualization.

## Ethics statement

This research was conducted under approval of the Mayo Clinic Institutional Review Board. This study was conducted in accordance with institutional ethical standards and the principles of the Declaration of Helsinki. All identifying information has been removed to protect patient privacy.

## Funding

None.

## Declaration of competing interest

The authors declare that they have no known competing financial interests or personal relationships that could have appeared to influence the work reported in this paper.
